# Emergency department use and geospatial variation in social determinants of health: a pilot study from South Carolina

**DOI:** 10.1186/s12889-023-16136-2

**Published:** 2023-08-11

**Authors:** Reid DeMass, Deeksha Gupta, Stella Self, Darin Thomas, Caroline Rudisill

**Affiliations:** 1 Department of Epidemiology and Biostatistics, Arnold School of Public Health, University of South Carolina, 915 Greene St., Columbia, SC 29208 USA; 2 Department of Health Promotion, Education and Behavior, Arnold School of Public Health, University of South Carolina, Columbia, SC 29208 USA; 3 Department of Epidemiology and Biostatistics, Arnold School of Public Health, University of South Carolina, 300 E. McBee Ave. Greenville, Columbia, SC 29601 USA; 4 Addiction Medicine Center, Prisma Health, 605 Grove Road Greenville, Columbia, SC 29605 USA; 5 Department of Health Promotion, Education and Behavior, Arnold School of Public Health, University of South Carolina, 300 E. McBee Ave. Greenville, Columbia, SC 29601 USA

**Keywords:** Social determinants of health, Healthcare resource use, Emergency department use, Geospatial variation, Spatially varying coefficient models

## Abstract

**Background:**

Health systems are increasingly addressing patients’ social determinants of health (SDoH)-related needs and investigating their effects on health resource use. SDoH needs vary geographically; however, little is known about how this geographic variation in SDoH needs impacts the relationship between SDoH needs and health resource use.

**Methods:**

This study uses data from a SDoH survey administered to a pilot patient population in a single health system and the electronic medical records of the surveyed patients to determine if the impact of SDoH needs on emergency department use varies geospatially at the US Census block group level. A Bayesian zero-inflated negative binomial model was used to determine if emergency department visits after SDoH screening varied across block groups. Additionally, the relationships between the number of emergency department visits and the response to each SDoH screening question was assessed using Bayesian negative binomial hurdle models with spatially varying coefficients following a conditional autoregressive (CAR) model at the census block group level.

**Results:**

Statistically important differences in emergency department visits after screening were found between block groups. Statistically important spatial variation was found in the association between patient responses to the questions concerning unhealthy home environments (e.g. mold, bugs/rodents, not enough air conditioning/heat) or domestic violence/abuse and the mean number of emergency department visits after the screen.

**Conclusions:**

Notable spatial variation was found in the relationships between screening positive for unhealthy home environments or domestic violence/abuse and emergency department use. Despite the limitation of a relatively small sample size, sensitivity analyses suggest spatially varying relationships between other SDoH-related needs and emergency department use.

**Supplementary Information:**

The online version contains supplementary material available at 10.1186/s12889-023-16136-2.

## Introduction

Social determinants of health (SDoH), the conditions in places where individuals live, study, and work [1], affect a range of health outcomes, including the use of healthcare services.

Individuals with SDoH needs such as food insecurity and housing instability have greater likelihood of emergency department (ED) visits, hospitalizations, and hospital readmissions than those without such needs [2–4]. Further, geographical features such as neighborhood characteristics and regional policies can influence an individual’s access to essential resources including healthcare services, education, and employment opportunities, and reinforce SDoH needs [5]. Health systems that do not have a process or mechanism to address the needs of the underserved bear a disproportionate burden of uncompensated care [6] and penalizations for frequent hospital readmissions [7]. Thus, to improve outcomes for the most vulnerable patients, health systems are increasingly addressing patient needs that fall outside of traditional health care through SDoH screening and clinical-community collaborations.

Health systems across the US have been implementing SDoH screening to identify socioeconomic and environmental factors affecting patients and to support care improvement and advocacy efforts [8]. They have also been collaborating with community-based organizations to intervene on these identified needs. However, despite the potential of SDoH screening and clinical-community collaborations to improve health outcomes (e.g., quality of life, diabetes and hypertension control, asthma exacerbation likelihood) and lower healthcare costs [9–13], there is limited understanding of how SDoH affects health resource use. Findings from prior studies have yielded conflicting evidence about the impact of SDoH-related interventions on ED visits [14–17]. However, studies have found SDOH-related interventions to reduce inpatient stays [17], and ambulatory care sensitive hospital admissions [16].

Individuals living in geographical areas where SDoH needs are not met experience a greater burden of conditions such as syphilis [18], HIV [19], cardiovascular disease [20]. In addition, individuals who live in socially-vulnerable areas are more likely to utilize emergency departments [14]. Ye et al. (2019) demonstrated that patients residing near hospitals or those living in zip codes with poor socioeconomic status have higher 90-day readmission rates than those living farther away and in higher socioeconomic status neighborhoods [21]. Similarly, Grunwell et al. (2022) identified census tract hotspots for pediatric intensive care unit admissions and readmissions for asthma and found that children living in these hotspots had poor socioeconomic status, lived in crowded households and lacked vehicles for transportation [22]. Evidence from these studies suggest community-level variation in health resources use due to SDoH needs. However, few studies have examined geospatial variation in the relationship between SDoH needs and health resource use using individual-level measures such as SDoH screening surveys. Few studies have examined the impact of SDoH as elicited by a screening tool. One such study is by Austin, Pappan, and Venkat (2021) who examined geographic variation in ED use related to remote SDoH screening for older patients during COVID-19 [23]. The authors found fewer ED visits among individuals residing in high socioeconomic status areas and receiving SDOH screening outreach than those who did not.

As in Austin, Pappan and Venkat (2021), our study focuses on ED utilization among populations with SDoH needs. Individuals with SDoH needs such as housing, food, transportation, and utilities are more likely to seek emergency care than those without such needs [24]. They are often unable to access primary care [25] due to financial constraints [26], lack of insurance [27], and lack of providers in local neighborhoods [28]. In fact, one study estimates that 13.7–27.1% of ED visits in the US could be prevented if individuals had access to primary care [29]. Lack of primary care exacerbates health conditions for which socially vulnerable individuals seek care in emergency medicine settings [30]. Moreover, almost 50% of inpatient admissions originate from emergency medicine care [31]. To comprehensively understand how individuals’ SDoH needs are associated with ED use, it is crucial to investigate this relationship in a geographical context.

This paper aims to understand the role of patients’ geospatial characteristics on healthcare resource use when piloting a program to screen for and intervene on SDoH needs within a large health system. We examine whether there is spatial variation in the association between responses to SDoH screening (e.g., food insecurity, housing instability, domestic safety, social isolation) questions piloted in a large health system and ED use after SDoH screening. Our findings will contribute to the current body of research investigating the influence of SDOH screening and intervention on ED utilization by elucidating how geographical factors affect this relationship.

## Data and methods

### Study sample

The study sample included Prisma Health patients, aged 18 years or older, engaged in ambulatory care and condition management, inpatient case management, or community health in South Carolina’s central (Midlands) and northwestern (Upstate) regions. This study population constitutes initial efforts to pilot the SDoH screening within the health system.

### Data sources

Prisma Health is the largest non-profit health system in South Carolina and treats about 1.4 million patients annually. Its geographic footprint covers about half of the state’s total population [32]. Census data based on the system’s geographic coverage describes the areas served by the health system as predominantly White (63.4%) with the second most common racial or ethnic group being Black (26.7%). Of individuals in this geographic area, 29.0% have a bachelor’s degree or higher (versus 32.9% nationally), 13.8% would be considered persons in poverty (versus 11.4% nationally) and 13.2% are without health insurance (versus 10.2% nationally). Median household income is $54,864 versus $64,994 nationally and areas covered have population density of 332.6 per square mile (< 500 per square mile and less than 2,500 people being rural) [32].

The data for this study came from the health system’s piloting of a digital SDoH screening and referral platform in 2020, called NowPow. NowPow is a software program, embedded within the electronic medical record (EMR), that matches patients with SDoH-related needs to local resources based on their demographic information (e.g., distance from home).

This study uses three data sources: SDoH screening information from NowPow, electronic medical record (EMR) data and geocoded patient addresses. This study was reviewed by the Institutional Review Board (IRB) at Prisma Health (IRB# Pro00105482) and approved as human subjects exempt. Informed consent was waived by the IRB committee, and the study was conducted in accordance to all relevant IRB guidelines.

Data was collected for the period June 1, 2019-December 31, 2020 from NowPow and two EMR sources. SDoH screening information comes from a 13-item SDoH screening questionnaire that draws from validated questions such as the Hunger Vital Sign [33], the UCLA Three-Item Loneliness Scale [34] and the Single Item Literacy Screener (SILS) [35]. The questionnaire has been further described elsewhere [36]. Questions and full responses appear in the Appendix 1. Questions were chosen to include those social needs exhibiting links to health outcomes and where resources were available to intervene locally. The questionnaire was verbally administered and captured SDoH needs for food insecurity, housing instability and quality, financial instability, transportation needs, interpersonal violence/abuse, language and health literacy, and social connectedness. Based on responses to screening questions, patients were referred to community-based resources via a digital prescription (‘HealtheRx’) in NowPow.

The two EMR data sources were the health system’s Epic and a local clinically integrated network’s database (inVio Health Network). A study database was created from NowPow questionnaire responses and EMR data by linking medical record numbers.

Multiple screens for a single patient could occur during the study period. For a given patient, the screen that took place earliest in the study period was categorized as the index screen. If multiple screens were recorded in a single day, the last screen of the day was categorized as the index screen.

Finally, patient addresses were available through the EMR. Each address was associated with a latitude–longitude from the United States Census Geocoder. The latitude–longitude coordinates were then linked to the 2020 TIGER Block Group Shapefile from the US Census Bureau.

#### Outcome variables

ED visit information was drawn from the inVio network EMR and the date of the index screen was available in the linked database. The number of ED visits after the index screen and before the end of the study period was calculated and was the primary outcome of interest in the study.

#### Key independent variable – SDoH screening responses

Responses to the SDoH screening questions from the 13-item questionnaire are modeled as follows. Eight of the thirteen screening questions were Yes/No, and ‘No’ was set as the reference level. Five questions included more than two response categories. Responses to these questions were dichotomized into Yes/No. For health literacy, ‘Never’ and ‘Rarely’ constituted the ‘No’ category and the remaining responses (‘Some of the time,’ ‘Often’, ‘Always’) were grouped into the ‘Yes’ category, as suggested by Morris et al. [35]. The remaining four questions (two concerning food insecurity and two concerning social connectedness) were categorized such that the response indicating the least frequency (e.g., ‘Never true’ or ‘Hardly ever’) was the ‘No’ category and the remaining responses were grouped into the ‘Yes’ category.

#### Covariates

Nine additional patient demographic variables were considered: smoking status (Yes/No), age at the time of the screen, female (Yes/No), pregnant (Yes/No), Hispanic (Yes/No), primary payer (Medicaid/Medicare/Other), race (White/Black/Other/Patient Refused), weight category according to BMI (Underweight/Healthy/Overweight/Obese), and total number of comorbidities (0–9).

To calculate the total number of comorbidities, the presence (present = 1, absent = 0) of each of asthma, cancer (heart, colorectal, prostate, lung, endometrial), chronic pulmonary disease, depression, diabetes, heart failure, substance use (alcohol use and drug use), Alzheimer’s disease (including related disorders or senile dementia), and cerebrovascular disease was summed. The Charlson Comorbidity Index [37] was used to define cerebrovascular disease; the remaining comorbidities were defined by the Chronic Conditions Data Warehouse (CCW) [38].

To create the weight category variable, BMI cutoffs were set at less than 18.5, 18.5 to less than 25, 25 to less than 30, and 30 or greater and corresponded to weight categories of underweight, healthy, overweight, and obese, respectively. The race data from the EMR included levels such as White, Black, American Indian or Alaskan Native, Asian, biracial or multiracial, etc. A new race variable was created that re-categorized levels to White, Black, Other, and Patient Refused. Race categories included Hispanic individuals and should not be interpreted as ‘non-Hispanic’.

### Analytical strategy

The number of ED visits after the index screen was the response variable and was modeled solely by a spatially varying intercept term. Bayesian zero-inflated Poisson, Poisson hurdle, zero-inflated negative binomial, and negative binomial hurdle regression models were considered. The zero-inflated negative binomial model was considered the best fit as it produced the smallest deviance criterion information (DIC). The model was used to identify if ED visits after the index screen per person per week varied across US Census block groups.

Next, the relationships between the number of ED visits and the response to each screening question were further explored with Bayesian zero-inflated Poisson, Poisson hurdle, zero-inflated negative binomial, and negative binomial hurdle models. Thirteen different models were created, one for each screening question. The expected value of the Poisson/negative binomial distribution governing the number of ED visits for individual $i$ was given by $\mathrm{exp}\left({\eta }_{i}\right)$, where ${\eta }_{i}={t}_{i}+ {{\varvec{x}}}_{{\varvec{i}}}{\varvec{\beta}}+{r}_{i}{\psi }_{{s}_{i}}$.

Here, ${t}_{i}$ is an offset term equal to the log of the number of weeks between individual $i$’s index screen and the end of the study period; ${{\varvec{x}}}_{{\varvec{i}}}$ is a vector of covariates belonging to individual $i$; ${\varvec{\beta}}$ is the associated vector of coefficients; ${r}_{i}=1$ if individual $i$ responded ‘yes’ to the screening question under consideration and 0 otherwise; ${s}_{i}$ denotes the census block group to which individual $i$ belongs; and ${\psi }_{s}$ is a spatially varying coefficient for block group $s$. The covariates initially included in ${{\varvec{x}}}_{{\varvec{i}}}$ were an intercept, smoking status, age at the time of the screen, female, pregnant, Hispanic, primary payer, race, weight category, and total number of comorbidities.

Models were fit using the INLA package version 22.05.07 [39] in R version 4.2.0 [40]. The vector of spatially varying coefficients ${\varvec{\psi}}=({\psi }_{1},{\psi }_{2}, \dots .,{\psi }_{S})\mathrm{^{\prime}}$ was assumed to follow a conditional autoregressive (CAR) model at the census block group level [41]. All parameters were assumed to follow the default prior specifications in the R INLA package for the ‘besagproper’ prior. For each model, zero-inflated Poisson, Poisson hurdle, zero-inflated negative binomial, and negative binomial hurdle regression were considered. The negative binomial hurdle model produced the lowest DIC for all 13 models and was designated as the model of choice. In addition to empirical support for the negative binomial hurdle model provided by DIC, the negative binomial hurdle model also has strong theoretical support. Negative binomial hurdle models are commonly used to model emergency department visits [42–45]. In a zero-inflated model, observations equal to zeros can either be ‘structural’ zeros (modeled by a point mass at zero) or ‘sampling’ zeros, modeled by the chosen count distribution. In a hurdle model, all zero observations are structural zeros and the count distribution models only non-zero observations. When analyzing healthcare visit data, which are likely to have high numbers of zeros, it is common to conceptualize individuals as first deciding to seek care and then determining (or being provided advice on) what kind or how much care to seek [46]. In this context, all zeros are structural zeros, as they arise from individuals who do not seek care, making the hurdle model more appropriate.

Next, backwards stepwise selection was performed on each model individually. Barring the intercept and spatial term, all other variables were considered for removal. A variable was removed if its exclusion lowered the model’s DIC by at least two. If multiple variables’ removal lowered the DIC past the threshold, the variable which decreased it to the greatest magnitude was removed. This process was repeated until no variable met the removal criterion, and the resulting model was used. The set of covariates included in the final model after the stepwise selection procedure varied by screening question. Table 1 indicates which covariates were included in each screening question model. Specifications were analyzed to assess whether the response to each screening question was associated with ED visits after the index screen differentially across block groups.

**Table 1:** Covariates included in each screening question model

Screening Question Model	Smoker	Age	Female	Preg-nant	His-panic	Primary Payer	Total Comorbidities	Race	Weight Category
1	x	x	x	x			x	x	x
2	x	x	x	x		x	x	x	x
3	x	x	x	x	x		x	x	x
4	x		x	x	x	x		x	x
5	x	x	x	x	x		x	x	x
6	x	x	x	x	x		x	x	x
7	x	x	x	x	x		x	x	
8	x	x	x	x	x		x	x	
9	x	x	x			x			
10	x	x	x		x	x	x		x
11	x	x	x	x	x		x	x	x
12	x	x	x	x	x		x	x	
13	x	x	x	x	x		x		

An ‘x’ indicates the variable was included in the model

## Results

### Study sample

Over the study period, 3,016 patient observations were recorded. Patient observations were dropped if they were non-index screenings (329, 10.9%). Of the 2,687 (89.1%) index screenings, 197 (7.3%) failed to geocode and were removed, while 2,490 (92.7%) were successfully linked to a latitude–longitude coordinate. Observations were removed if the addresses were outside of South Carolina (7, 0.3%); had an obvious address miscoding (e.g., the address on file was ‘Missing’ but was still assigned to a latitude-longitude; 32, 1.2%); were duplicate observations (12, 0.4%); or belonged to a county for which less than 50% of block groups had observations (244, 9.1%). Ten counties met the final criterion above. In total, 2,195 observations were used for analysis (81.7% of the total index screen observations).

#### Descriptive statistics of patients

Descriptive statistics for the 2,195 patients are reported in Table 2. The number of ED visits after the screen variable was zero-inflated and positively skewed with a mean of 0.343 and a median of zero. The mean patient age was 63.1 ± 14.7 years and most patients were categorized as obese (54.1%). Majority patients were female (63.1%), White (52.8%), and enrolled in Medicare as their primary payer (61.2%). The most common comorbidities included diabetes (47.3%), depression (33.8%), and heart failure (29.2%), and the mean number of comorbidities per patient was 2.1 ± 1.7. The mean number of ED visits after the screen was 0.343 ± 1.535. The screening questions with the highest positive response rates were Questions 1 (11.5%), 2 (9.9%), 11 (11.7%), and 12 (6.0%), capturing SDoH needs for food insecurity, health literacy, and social connectedness, respectively.

**Table 2:** Patient demographics and responses to screening questions

Variables	N	Mean/ Proportion	Std. Dev	Median*
***Demographic variables***				
ED visits after the screen	2195	0.343	1.535	0
BMI Category	1929			
Underweight	34	0.018	0.003	
Healthy	358	0.186	0.009	
Overweight	494	0.256	0.010	
Obese	1043	0.541	0.011	
Age	2195	63.05	14.73	63.0
Female	2195	0.631	0.010	
Race/ethnicity	2195			
White	1159	0.528	0.011	
Black	824	0.375	0.010	
Other	210	0.096	0.006	
Patient refused	2	0.001	0.001	
Hispanic	2195	0.027	0.003	
Primary payer	1076			
Medicaid	59	0.055	0.007	
Medicare	658	0.612	0.015	
Other	359	0.334	0.014	
Smoking status (Yes)	2195	0.173	0.008	
Pregnancy status at the time of screen	2195	0.009	0.002	
***Comorbidities***	2195			
Diabetes	1038	0.473	0.011	
Depression	742	0.338	0.010	
Heart Failure (HF)	642	0.292	0.010	
Chronic obstructive pulmonary disease (COPD)	597	0.272	0.009	
Coronary vascular disease (CVD)	410	0.187	0.008	
Asthma	361	0.164	0.008	
Substance use disorder	339	0.154	0.008	
Cancer	251	0.114	0.007	
Alzheimer’s	216	0.098	0.006	
Total number of comorbidities		2.094	1.669	2
***Responses to screening questions (dichotomized)***				
Q1. Within the past 12 months we worried whether our food would run out before we got money to buy more				
No	1928	0.878	0.007	
Yes	252	0.115	0.007	
No response	15	0.007	0.002	
Q2. Within the past 12 months the food we bought just didn't last and we didn't have money to get more				
No	1922	0.876	0.007	
Yes	218	0.099	0.006	
No response	55	0.025	0.003	
Q3. Are you worried that in the next 3 months, you may not have a safe or stable place to live? (risk of eviction, being kicked out, homelessness)				
No	2054	0.936	0.005	
Yes	79	0.036	0.004	
No response	62	0.028	0.004	
Q4. Are you worried that the place you are living now is making you sick? (has mold, bugs/rodents, water leaks, not enough heat or air conditioning)?				
No	2068	0.942	0.005	
Yes	47	0.021	0.003	
No response	80	0.036	0.004	
Q5. In the past 3 months, has the electric, gas, oil or water company threatened to shut off services to your home?				
No	2006	0.914	0.006	
Yes	89	0.041	0.004	
No response	100	0.046	0.004	
Q6. In the past 3 months, has lack of transportation kept you from medical appointments or getting your medications?				
No	2054	0.936	0.005	
Yes	79	0.036	0.004	
No response	62	0.028	0.004	
Q7. Was there a time in the past 3 months when you needed to see a doctor or buy medications but could not because of cost?				
No	1941	0.884	0.007	
Yes	131	0.060	0.005	
No response	123	0.056	0.005	
Q8. Are you finding it so hard to get along with a partner, spouse, or family members that it is causing you stress?				
No	1976	0.900	0.006	
Yes	57	0.026	0.003	
No response	162	0.073	0.006	
Q9. Does anyone in your life hurt you, threaten you, frighten you or make you feel unsafe?				
No	2004	0.913	0.006	
Yes	14	0.006	0.002	
No response	177	0.081	0.006	
Q10. Do you want help with education options, learning English, or job training for yourself?				
No	1986	0.905	0.006	
Yes	34	0.015	0.003	
No response	175	0.080	0.006	
Q11. How often do you need to have someone help you when you read instructions, pamphlets, or other written material from your doctor or pharmacy?				
No	1775	0.809	0.008	
Yes	256	0.117	0.007	
No response	164	0.075	0.006	
Q12. How often do you feel that you lack companionship?				
No	1853	0.844	0.008	
Yes	132	0.060	0.005	
No response	210	0.096	0.006	
Q13. How often do you feel left out?				
No	1867	0.851	0.008	
Yes	113	0.051	0.005	
No response	215	0.098	0.006	

^*^Median calculated for continuous/count variables only

Table 3 reports the mean number of ED visits per person per week for each demographic variable and response to screening question.

**Table 3:** Mean number of emergency department visits per week stratified by patient demographics and responses to screening questions

Variables	Mean	Std. Dev	*P*-value*
***Demographic variables***			
BMI Category			0.020
Underweight	0.078	0.347	
Healthy	0.033	0.124	
Overweight	0.027	0.102	
Obese	0.022	0.141	
Age			0.904
< 65	0.021	0.115	
65 or older	0.027	0.142	
Female			0.646
Yes	0.030	0.144	
No	0.021	0.118	
Race/ethnicity			0.031
White	0.028	0.150	
Black	0.023	0.108	
Other	0.009	0.042	
Patient refused	0.000	0.000	
Hispanic			0.070
Yes	0.022	0.086	
No	0.024	0.129	
Primary payer			0.017
Medicaid	0.022	0.096	
Medicare	0.034	0.169	
Other	0.023	0.119	
Smoking status			0.014
Yes	0.040	0.147	
No	0.021	0.124	
Pregnancy status at the time of screen			
Yes	0.007	0.033	0.531
No	0.024	0.129	
***Comorbidities***			
Diabetes			< 0.001
Yes	0.032	0.154	
No	0.017	0.099	
Depression			< 0.001
Yes	0.039	0.179	
No	0.017	0.091	
Heart Failure			< 0.001
Yes	0.047	0.188	
No	0.015	0.092	
COPD			< 0.001
Yes	0.045	0.184	
No	0.017	0.099	
CVD			0.002
Yes	0.043	0.211	
No	0.020	0.100	
Asthma			0.167
Yes	0.030	0.125	
No	0.023	0.129	
Substance use disorder			< 0.001
Yes	0.062	0.209	
No	0.017	0.106	
Cancer			0.002
Yes	0.040	0.166	
No	0.022	0.122	
Alzheimer’s			0.005
Yes	0.037	0.122	
No	0.023	0.129	
***Responses to screening questions (dichotomized)***			
Q1. Within the past 12 months we worried whether our food would run out before we got money to buy more			0.032
No	0.024	0.131	
Yes	0.026	0.106	
No response	0.004	0.014	
Q2. Within the past 12 months the food we bought just didn't last and we didn't have money to get more			0.026
No	0.025	0.133	
Yes	0.023	0.093	
No response	0.016	0.093	
Q3. Are you worried that in the next 3 months, you may not have a safe or stable place to live? (risk of eviction, being kicked out, homelessness)			0.301
No	0.025	0.131	
Yes	0.022	0.073	
No response	0.014	0.088	
Q4. Are you worried that the place you are living now is making you sick? (has mold, bugs/rodents, water leaks, not enough heat or air conditioning)?			0.415
No	0.023	0.121	
Yes	0.067	0.302	
No response	0.032	0.139	
Q5. In the past 3 months, has the electric, gas, oil or water company threatened to shut off services to your home?			0.086
No	0.024	0.217	
Yes	0.021	0.074	
No response	0.039	0.217	
Q6. In the past 3 months, has lack of transportation kept you from medical appointments or getting your medications?			0.301
No	0.025	0.131	
Yes	0.022	0.073	
No response	0.014	0.088	
Q7. Was there a time in the past 3 months when you needed to see a doctor or buy medications but could not because of cost?			0.082
No	0.024	0.125	
Yes	0.018	0.054	
No response	0.031	0.196	
Q8. Are you finding it so hard to get along with a partner, spouse, or family members that it is causing you stress?			0.076
No	0.024	0.125	
Yes	0.023	0.064	
No response	0.027	0.174	
Q9. Does anyone in your life hurt you, threaten you, frighten you or make you feel unsafe?			0.172
No	0.024	0.123	
Yes	0.101	0.270	
No response	0.024	0.164	
Q10. Do you want help with education options, learning English, or job training for yourself?			0.736
No	0.024	0.123	
Yes	0.023	0.084	
No response	0.030	0.084	
Q11. How often do you need to have someone help you when you read instructions, pamphlets, or other written material from your doctor or pharmacy?			0.778
No	0.024	0.186	
Yes	0.020	0.090	
No response	0.032	0.186	
Q12. How often do you feel that you lack companionship?			0.590
No	0.025	0.127	
Yes	0.016	0.059	
No response	0.027	0.165	
Q13. How often do you feel left out?			0.665
No	0.025	0.128	
Yes	0.022	0.084	
No response	0.022	0.150	

^*^P-values from Kruskal–Wallis analysis

Table 3 demonstrates that there are statistically significant differences between ED visits per week after the index screen between levels of a given variable as evidenced by Kruskal–Wallis analyses. Smoking status, primary payer, race/ethnicity, and categorized BMI are all statistically significant demographic variables. Notably, asthma is the only comorbidity that is not statistically significant. Among the SDoH needs, both the screening questions on food insecurity, questions one and two, were found to be statistically significant.

### ED visits across block groups

**Fig. 1:**
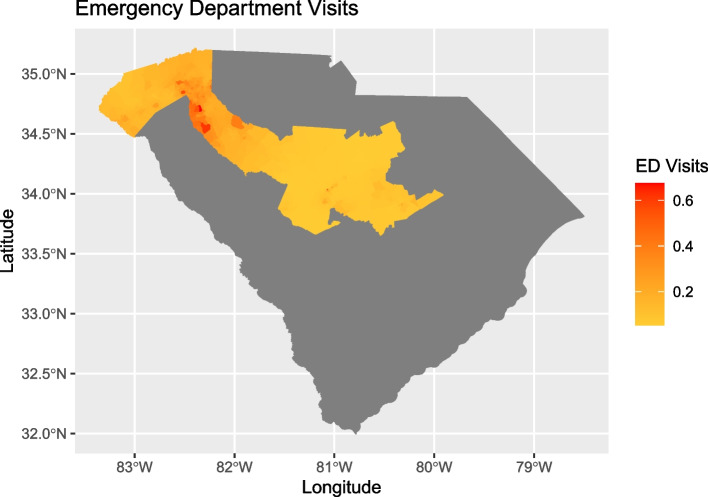
Mean ED visits per person per week after index screen at block group level

The predicted mean number of ED visits per person per week ranged from 0.051 to 0.718 across block groups. Statistically important differences in ED visits were found between block groups. A pair of block groups were considered to have statistically important differences if the 95% credible intervals estimating the mean number of ED visits per person per week from each block group did not overlap. Figure 1 shows the posterior mean estimate of the mean number of ED visits per person for each block group for the 10 counties from which data was drawn. The darker coloration denotes a greater mean number of ED visits per person per week.

### Relationship between ED visits and response to screening questions

Statistically important spatial variation was found in the association between the mean number of ED visits after the screen and patient response to question four (‘Are you worried that the place you are living now is making you sick? (has mold, bugs/rodents, water leaks, not enough heat or air conditioning)?’) as well as question nine (‘Does anyone in your life hurt you, threaten you, frighten you or make you feel unsafe?’; screener for violence/abuse). No other questions exhibited statistically important spatial variation in association with the mean number of ED visits. Two block groups were considered to have statistically important variation in association with mean number of ED visits if the 95% credible intervals for the spatially varying coefficient of the response to the screening question did not overlap.

Based on the model for response to question four, the mean number of ED visits per week for individuals who answered ‘Yes’ ranged from 0.451 to 7.87 times the mean number of visits per week for individuals who answered no. For the model based on question nine, the range was 0.430 to 6.327. Of the 1,110 block groups contained in the 10 counties, the 95% credible intervals for the Incident Rate Ratio (IRR) were entirely above one for 30 and 28 block groups for question four and nine models, respectively. Furthermore, 27 block groups had IRR intervals that were entirely above one for both question four and nine. For each model, all remaining block groups contained one in the interval; no interval was entirely below one for any block group.

**Fig. 2:**
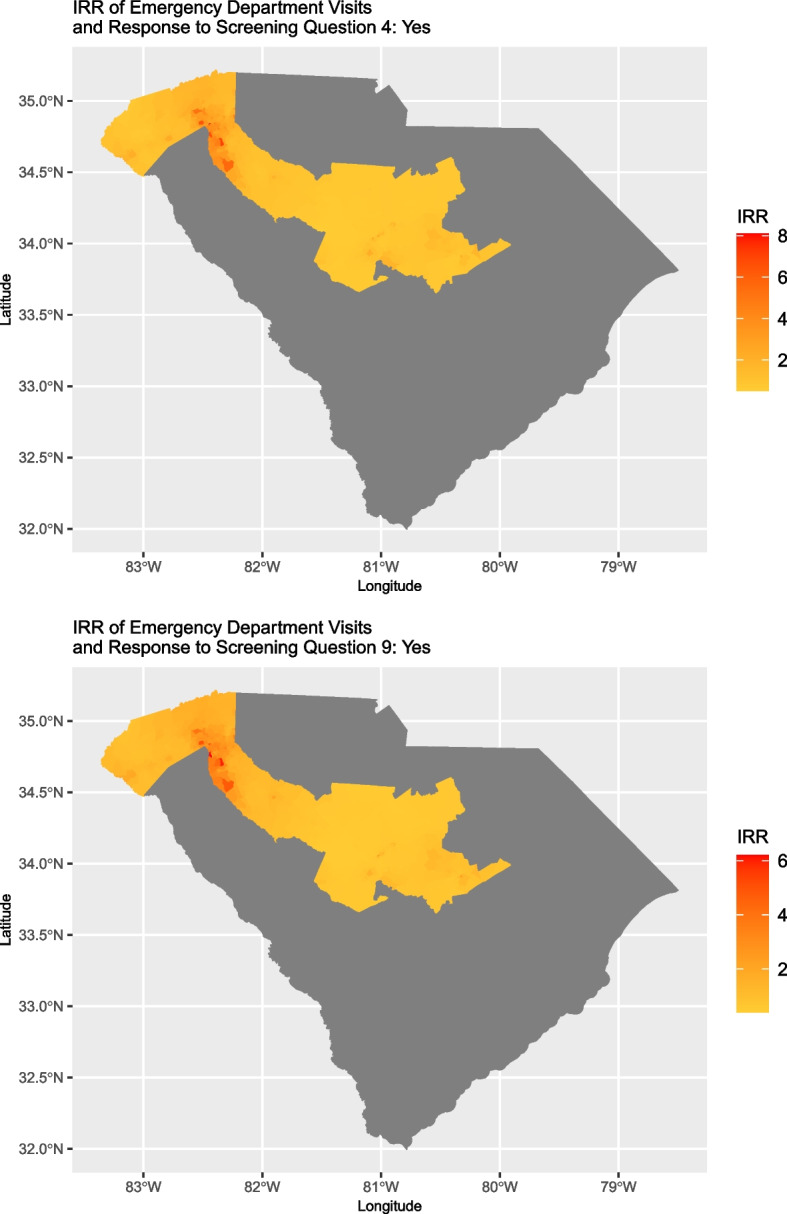
Incident Rate Ratio (IRR) estimates for the mean number of ED visits per week for individuals who answered ‘Yes’ to screening questions four, unhealthy home environment (top) or nine, domestic violence/abuse (bottom); darker coloration denotes larger IRRs

**Fig. 3:**
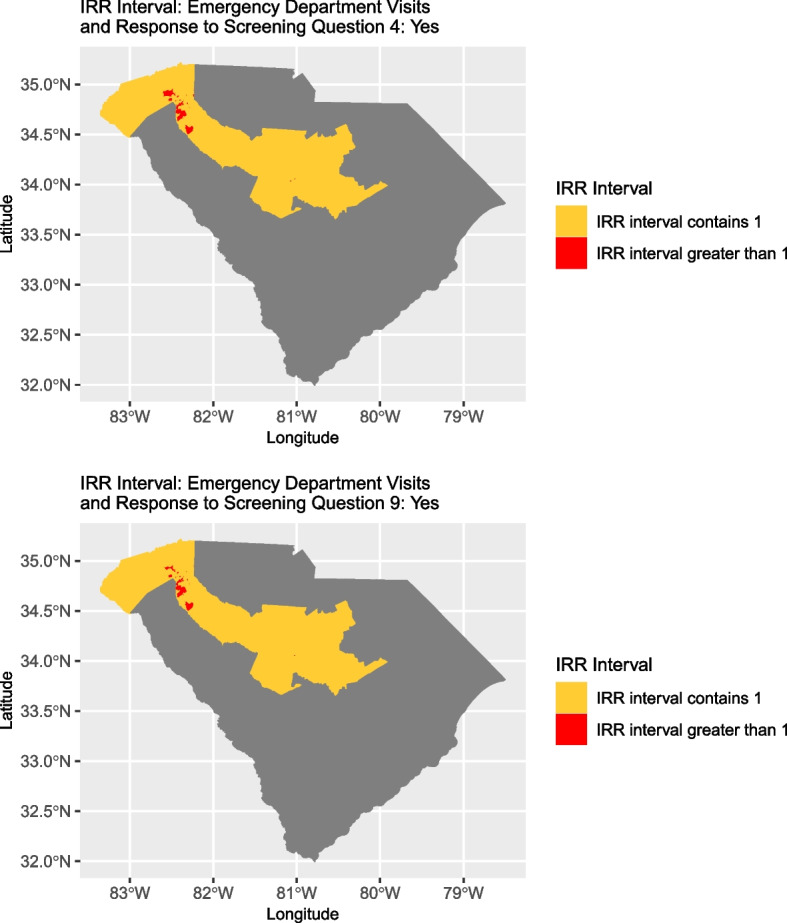
The location of block groups for which the estimate interval associated with answering positively to questions four, unhealthy home environment (top), or nine, domestic violence/abuse (bottom), was entirely above one (denoted by darker coloration)

Figure 2 shows the estimates of the IRR for the mean number of ED visits per week for individuals who answered ‘Yes’ to screening question nine and Fig. 3 shows the location of block groups for which the estimate interval was entirely above one.

Table 4 details the estimates for all variables in the question four and nine models. For both models, female patients were found to have significantly lower number of ED visits after the index screen ( $\beta$= -0.303; 95% credible interval: [-0.605, -0.001] for question four; $\beta$ = -0.361; 95% credible interval: [-0.659, -0.063] for question nine), while patients who smoke were found to have significantly higher number of ED visits after the index screen ($\beta$ = 0.355; 95% credible interval: [0.009, 0.703] for question four; $\beta$ = 0.477; 95% credible interval: [0.137, 0.824] for question nine). No other covariates were found to have a statistically important association with the mean number of ED visits after the screen. Statistical importance was defined as having a 95% credible interval which did not contain zero.

**Table 4:** Fixed effects (${\varvec{\beta}}{\varvec{s}})$ for regression models predicting mean number of ED visits per week after index screen from demographic variables and response to screening questions four or nine

	**Question 4, unhealthy home environments**			**Question 9 domestic violence/abuse**		
**Effect**	**Mean**	**0.025 Quantile**	**0.975 Quantile**	**Mean**	**0.025 Quantile**	**0.975 Quantile**
Constant (intercept)	-2.652	-3.656	-1.644	-2.851	-4.050	-1.696
Tobacco use	0.355	0.009	0.703	0.477	0.137	0.824
Age	NA	NA	NA	0.006	-0.005	0.017
Female	-0.303	-0.605	-0.001	-0.361	-0.659	-0.063
Primary Payer: Medicare	0.707	-0.197	1.639	0.464	-0.344	1.286
Primary Payer: Missing	0.460	-0.454	1.354	0.308	-0.546	1.170
Primary Payer: Other	0.357	-0.588	1.301	0.342	-0.488	1.167
Race: White	0.108	-0.259	0.470	NA	NA	NA
Race: Other	-0.490	-1.134	0.152	NA	NA	NA
Race: Patient Refused	-8.445	-34.570	17.695	NA	NA	NA
Weight: Underweight	0.114	-0.715	0.956	NA	NA	NA
Weight: Overweight	0.007	-0.442	0.453	NA	NA	NA
Weight: Obese	-0.192	-0.568	0.172	NA	NA	NA
Weight: Missing	-0.477	-1.123	0.162	NA	NA	NA

Note: ‘NA’ signifies that this variable does not appear in the model because of stepwise selection procedure

### Sensitivity analyses

As a robustness check, the models for each screening question were rerun with the total number of ED visits over the study period as the response variable, instead of number of ED visits after screening. The ‘total number of ED visits’ variable is the count of the number of ED visits for a given patient over the entire study period. This variable might capture ED use more accurately as it contains information for a longer time period (19 months). Additionally, it is less zero-inflated as compared to the ‘ED visits after the screen’ variable. However, the drawback of ‘total number of ED visits’ is that a patient’s SDoH needs were unknown for the entirety of this time period. SDoH needs are only known after the screening questionnaire was administered. However, neither question four nor nine includes any kind of time reference such as ‘in the last 3 months’ or ‘within the past 12 months’ when asking for recall as do some SDoH questions in the screening questionnaire. Therefore, responses could be referencing the current time, very immediate past or a longer time frame of episodic or continued circumstances.

Despite this limitation, the total ED use variable was modeled for each of the 13 screening questions to understand further the implications of choice in modeling ED use. The covariates used in each model were identical to those used in the models predicting ED use after the screen; however, the offset term was removed as the ‘total number of ED visits’ response was collected over the same time period for all individuals. Interestingly, statistically important spatial variation was found for all 13 questions. Again, spatial variation was statistically important if at least two block groups’ 95% credible intervals for the spatial coefficient of screening question response did not overlap.

Various methods for model selection exist, with stepwise procedures being among the most common. To assess the sensitivity of our findings to the proposed backwards stepwise model selection approach, forward selection was also performed for the models involving questions four and nine. After forward selection, the model for question four contained smoker and primary payer while the model for question nine contained smoker, age and Hispanic. Both models exhibited significant spatial variation in the relationship between screening positive and the ED visits, indicating that our findings are robust to the method of model selection. As a final check, models were fit for questions four and nine which had all variables found to be significantly related to total ED visits in Table 3 (weight category, race, primary payer, smoker and total number of comorbidities). These models also exhibited significant spatial variation in the relationship between screening positive and ED visits.

Spatial patterns in health services are nearly always due to spatial patterns in underlying drivers or mediators of health service use, rather than to the direct effect of latitude and longitude. To further investigate which factors might be contributing to the spatially varying relationship between screening positive for an unhealthy home environment or domestic violence and ED use, we performed a sensitivity analysis in which the ‘number of comorbidities’ variable was replaced with nine binary variables indicating the presence or absence of each of the comorbidities. For question four, backwards stepwise elimination resulted in a model which contained smoker, age, female, pregnancy status, Hispanic, primary payer, diabetes, COPD, CVD, cancer, CHF, depression, substance abuse disorder and Alzheimer’s disease. This model exhibited significant spatial variation in the relationship between living in an unhealthy home and ED visits. For question nine, the final model contained the following variables: smoker, age, female, pregnancy status, Hispanic, primary payer, race, BMI category, COPD, CVD, cancer, CHF, depression, substance use disorder, Alzheimer’s disease, and asthma. Notably, there was no longer significant spatial variation in the effect of domestic violence on ED visits in this model. This suggests that spatial patterns in one or more of the comorbidities in the model are contributing to the spatial variation in the effect of domestic violence on ED use.

## Discussion

This study examines whether ED use after SDoH screening is associated with identified SDoH needs and geographic locations. This analysis uses pilot data from an initial pilot rollout of SDoH screening at the largest health system in South Carolina.

Our findings indicate that ED visits varied across block groups. Within some block groups, the likelihood of ED visits after screening was greater among those who lived in an unhealthy home or screened positive for violence/abuse. However, for other block groups no significant differences in ED visits were observed among those experiencing these SDoH-related needs and those who were not. Finally, the likelihood of ED visits after screening among those who screened positive for an unhealthy home environment or violence/abuse was found to vary across block groups.

Likelihood of ED visits after screening among those reporting an unhealthy home environment (mold, bugs/rodents, water leaks, not enough heat or air conditioning) remained robust under a number of sensitivity checks. This supports previous research finding a link between housing quality and ED visit likelihood for specific conditions such as asthma [47]. However, the fact that we still find spatial variation after accounting for individual comorbidities suggests that there are more factors than individual comorbidities predicting the impact of spatial variation in unhealthy home environments in ED visits. Previous work examining housing quality in census tracts has also found an association with likelihood of ED visits for asthma among Hispanics/Latinos [48] as well as housing quality and ED use among children [49]. The screener used in our study has another question specifically on housing instability, which interestingly, was not significant for spatial variation in impacting ED visit likelihood. This is surprising given the frequency of housing needs seen in the EDs [50] and findings about increased likelihood of ED visits with housing instability [51]. These findings suggest that on a neighborhood basis, intervening on housing quality is an important policy consideration.

Interestingly, spatial variation vanished when specific comorbidities were included in the model for violence/abuse. This suggests that spatial patterns in comorbidities may be mediating the effects of violence/abuse on ED use. Future studies should examine this relationship more closely to include specific comorbidities that might be more likely to affect populations with violence/abuse such as anxiety, injury and chronic pain [52]. Existing studies have found greater ED utilization among individuals experiencing physical violence/abuse than those never experiencing violence/abuse [53], warranting screening within health systems’ emergency care services. However, like our study, existing research has limited evidence for the impact of SDoH screening on ED use for this specific population. Specifically, studies have shown that violence/abuse screening does not reduce the risk of violence/abuse post screening [54, 55] and has no differential effect on ED use [56].

### Limitations

This work has a number of limitations deserving mention. First, this study took place during spring 2020, when COVID-19 impacted ED use with a general decline in use [57] but an increase among patients experiencing violence/abuse [58]. Second, as this is pilot work, the study population is not generalizable to patients outside of ambulatory care and condition management, inpatient case management, or community health programs. Third, the sample size is small which may impact the study power. However, our sample’s food insecurity level is in line with food insecurity rates in South Carolina [59] indicating the potential representativeness of SDoH needs in our sample despite the sub-group representation of clinical patients and small sample size. Fourth, our study includes only nine months of ED use data after screening which may not be sufficient to see changes in health resource use, especially among violence/abuse victims. Use of referral services may vary based on victims’ readiness for implementing changes towards safety. This process is often non-linear [60] and may involve multiple attempts over a long period of time [61]. Future studies should examine geospatial variation in health resource use over longer duration.

A notable limitation is the small number of patients who screened positive for violence/abuse (*n* = 14, 0.64%). While we expect this SDoH question to have a low percentage positive rate relative to more common SDoH needs such as food insecurity, the total study sample size means that the count of positive screens is a small number. This sample size for violence/abuse calls into question that specific sub-group's representativeness of the larger population who possess SDoH needs for violence/abuse. Furthermore, such a small positive screening rate limits confidence in the model accurately capturing the true spatial variation across block groups of the association between violence/abuse and ED use after the screen. Positively however, each patient who screened positive for violence/abuse resided in a distinct block group allowing the model to leverage as much spatial information as possible which contributes to robustness.

Furthermore, screening questions four and nine on unhealthy home environments and violence/abuse had the third lowest and first lowest positive screening rate (respectively) out of all 13 SDoH questions. Assuming true spatial variation was present in the underlying population, it would be expected that screening questions with larger positive screening rates would lend themselves to greater model power and pick up on statistically important spatial variation, Therefore, it is somewhat surprising that only these two screening questions were found to indicate statistically important spatial variation. Although we only found significant spatial variation in ED use for responses regarding unhealthy home environments and violence/abuse, our study warrants future research to examine spatial variation of health resource use based on other SDoH needs with a larger sample size, greater number of positive screens for a given SDoH need, longer data collection time periods, and individuals captured from a wide distribution of locations. Moreover, our brief robustness checks indicate that health system resource use data that is less zero-inflated might better perform for similarly designed spatial models. Our findings regarding unhealthy home environments and violence/abuse being the only SDoH questions to exhibit spatial variation in ED use could be the result of small sample size and the highly zero-inflated nature of the ED use after the screen count variable. Thus, this point alone should not exclude the potential importance of geospatial variation with other SDoH needs and health resource use, especially given existing knowledge about how SDoH needs have a clear geospatial component. This remains a question of further study as SDoH screening becomes more widespread in health systems.

### Policy implications

Use of geospatial analysis of health resource use has several policy implications. Integrating geospatial information with EHR data can help health systems identify vulnerable communities and “hotspots” for health resource use. Identifying communities with the greatest healthcare needs associated with SDoH can facilitate clinical-community efforts in addressing such needs through appropriate resource provision. It also enables health systems to prioritize care and resources towards its most vulnerable patients and implement targeted SDoH-related interventions in the community where people live. Such targeted interventions can help health systems improve patient health outcomes and quality of life, and reduce costs and penalties associated with inappropriate health resource utilization.

### Supplementary Information

**Additional file 1. **Appendix

## Data Availability

The data that support the findings of this study are available from Prisma Health, but restrictions apply to the availability of these data, which were used under agreement for the current study, and so are not publicly available. Deidentified data are however available from the authors upon reasonable request and with permission of Prisma Health.

## Abbreviations

SDoH: Social Determinants of Health
ED: Emergency Department
US: United States
EMR: Electronic Medical Record
IRR: Incidence Rate Ratio
IRB: Institutional Review Board
