# Supplementary material for: Emergency department use and geospatial variation in social determinants of health: a pilot study from South Carolina
Source: BMC Public Health. 2023 Aug 11;23:1527. doi: 10.1186/s12889-023-16136-2 (PMC10416539; doi:10.1186/s12889-023-16136-2)
Supplement: Supplementary file 1 — Additional file 1. Appendix [file 12889_2023_16136_MOESM1_ESM.docx]

**Appendix 1. 13-questions in the SDoH screening survey**

| **SDoH domains** | **Screening Questions** | **Responses** |
| --- | --- | --- |
| Food insecurity | Within the past 12 months we worried whether our food would run out before we got money to buy more. | Often true, Sometimes true, Never true |
|  | Within the past 12 months the food we bought just didn't last and we didn't have money to get more. | Often true, Sometimes true, Never true |
| Housing instability/quality | Are you worried that in the next 3 months, you may not have a safe or stable place to live? (risk of eviction, being kicked out, homelessness) | Yes, No |
|  | Are you worried that the place you are living now is making you sick? (has mold, bugs/rodents, water leaks, not enough heat or air conditioning) | Yes, No |
| Utilities insecurity | In the past 3 months, has the electric, gas, oil or water company threatened to shut off services to your home? | Yes, No |
| Lack of transportation | In the past 3 months, has lack of transportation kept you from medical appointments or getting your medications? | Yes, No |
| Financial instability | Was there a time in the past 3 months when you needed to see a doctor or buy medications but could not because of cost? | Yes, No |
| Violence/abuse | Are you finding it so hard to get along with a partner, spouse, or family members that it is causing you stress? | Yes, No |
|  | Does anyone in your life hurt you, threaten you, frighten you or make you feel unsafe? | Yes, No |
| Language or educational needs | Do you want help with education options, learning English, or job training for yourself? | Yes, No |
|  | How often do you need to have someone help you when you read instructions, pamphlets, or other written material from your doctor or pharmacy? | Never, Rarely, Some of the time, Often, Always |
| Social connectedness | How often do you feel that you lack companionship? | Hardly ever, Some of the time, Often |
|  | How often do you feel left out? | Hardly ever, Some of the time, Often |
